# The preferred role of nurses in family physician team

**DOI:** 10.3934/publichealth.2020018

**Published:** 2020-03-27

**Authors:** Hesamedin Askari-Majdabadi, Sefollah Alaei, Nastaran Jafarian, Habiballah Safari, Hossein Habibian

**Affiliations:** 1 Nursing Care Research Center, Semnann University of Medical Sciences, Semnan, Iran; 2 Treatment Affairs Deputy, Semnan University of Medical Sciences, Semnan, Iran; 3 Health Deputy, Semnan University of Medical Sciences, Semnan, Iran; 4 Health Deputy, Health Statistics Unit, Semnan University of Medical Sciences, Semnan, Iran

**Keywords:** nurses, patient care team, family physician's team, attitude of health personnel

## Abstract

**Introduction:**

Nurses are considered as one of the members of family physician teams, therefore it is important to explain the role of nurses in it. The aim of study was to determination of nurses' roles in the family physician team.

**Methods:**

This research was conducted as a cross sectional descriptive analytical study. The study population was consisted of all the nurses who were working in under the supervision of Semnan University of Medical Sciences. The participants included 150 nurses who were selected via random stratified sampling method. The research instrument was a researcher-made questionnaire.

**Results:**

Of all the participants, 30 persons (20%) were male and the rest were female (80%). The findings of the study reported based on the mean score of five. The collected data indicated that the nurses participating in the study, as a member of family physician team, had a tendency to take part in the following activities: support (4.44), care (4.37), management (4.05), counseling (3.93), prevention (3.81), and education (3.78).

**Conclusion:**

The results of the study showed that the nurses were more interested in playing a role in supporting and caring for patients, rather than participating in education and preventive activities. Since family physician teams must mainly provide community-based preventive services, the authorities should try to enhance the interests and capabilities of nurses to empower them to play a role in preventive and education activities.

## 1. Introduction

Family physician team is responsible for the delivery of comprehensive care services for individuals and families of different age and sex [1]. The performance of family physician depends on team's familiarity with the patient and the community and it puts emphasis on the prevention of diseases and promotion of health. As defined by the WHO, the main goal of the family physician plan is to provide comprehensive and continuous care for people, with a special focus on the family and community [2]. A family physician in the United States is considered as a specialty, ranked after general medicine [3]. In Iran, Tehran University of Medical Sciences has introduced a virtual specialized course on master of public health (MPH) and family physician [4].

It is worth noting that, the presence of manpower with the appropriate expertise is an essential requirement for the design of every organization [5],[6]. When teams are used for implementing health care activities, complex problems and major health issues can be addressed properly, because when a team is formed, the resources, talents, and skills of various members of the group can be utilized [7]–[12]. In a family physician team, in addition to general practitioners, there are some other main members including nurses and midwives who are also considered to be the permanent members of the team [5]. The results of previous studies show that, nurses are the only professional groups that have a comprehensive approach toward fulfilling the needs of patients and service recipients [13]. The membership of nurses in the family physician team is one of the few cases that have been considered and addressed in planning and design of organizational structure of community-based services [14]. However, the successful participation of the nursing staff in the program depends on the nurses' clarify role in membership of the team and their commitment in playing their own roles properly. On the other hand, relevant organizations also should provide the nurses with the necessary support to motivate them paly their roles [15].

Despite the emphasis of the national development plans on the implementation of the family physician program, it is launched in urban areas with a delay. The program is fully implemented only in two provinces of Fars and Mazandaran, which were accompanied with the relative satisfaction of service recipients [16]. Unfortunately, the role of nurses in the team is not precisely defined, and the executive guideline of the family physician plan uses the words “nurse or midwife” when defining the positions and roles in the team [14]. Nurses can play diverse and effective roles at three levels of prevention [17]. Nurses' role defined by International Council of Nurses (ICN) as “encompasses autonomous” and collaborative care of individuals of all ages, families, groups and communities, sick or well and in all settings. Nursing includes the promotion of health, prevention of illness, and the care of ill, disabled and dying people. Advocacy, promotion of a safe environment, research, participation in shaping health policy and in-patient care and health systems management, and education are also key nursing roles [18]. However, clarification of positions and roles is important for organizing human resources and teamwork [19] and it is necessity to focus on all members of family physician team [20]. Few studies have been carried out on the role of nurses in the family physician team [21]. In a historical point of view, the role of nurses in the family physician team is not defined based on professional qualifications, on the contrary, it is defined based on the needs and the existing tasks and duties [22]. However, there are problems with the structure and performance of these health service provider teams. For example, despite the fact that the family physician program has been implemented in Eastern Europe for about a decade, the position and the role of the team members are not well defined [23]. The results of another study shows that, because of the lack of defined positions and task descriptions, the nurses in the family physician team plays a wide range of roles, from a receptionist to a primary care service provider [24].

Obviously, in order to make coordination in a group, it is of great importance to define and accept the role of any individual in the group; the position and role of each team member must be accepted by the person taking the role, as well as by other individuals in the team. Therefore, since the presence of nurses in the family physician, team is a new experience in developing country's health systems and for the nursing staff as well, conducting new studies can help to provide a more accurate explanation of the status and position of the team members, including nurses, and can provide a ground for achieving success. Thus, this study was conducted to determine the preference of nurses about their role in family physician team. In addition to personal interests and organizational variables, it seems other variables such as gender and work experience could be influential in the tendency to different roles of nurses. Therefore, the effect of these factors on the preferential role of nurses in the family physician team has also been considered.

## 2. Materials and methods

### 2.1. Study design

This research was conducted as a cross sectional descriptive analytical study in 2018 to determination nurses' roles in the family physician team by participatory approach. The study population was consisted of all the nurses with at least the bachelor degree in nursing who were working in the health care centers affiliated to Semnan University of Medical Sciences, public (governmental), semi-public, and private centers.

### 2.2. Sample size and sampling method

The study population also included the nurses who were not employed but were a member of professional organizations such as the Nursing Organization. The statistical study population was consisted of 400 nurses. Using the Cochran formula with a precision of 5% and Z = 1.96, the sample size was calculated as 196 people. The subjects of the research were selected via stratified sampling method. The nurses under the study were distributed and working in four different areas, including the followings: Amiralmomenin Hospital, Kosar Hospital, Panzdah Khordad Hospital, as well as the private sector and outside the above mentioned places. At first, the population ratio of each area was determined and then the subjects were randomly selected from the community in proportion to the population working in each area, without reconstitution. Participants in the study were autonomous to complete the questionnaire, but they were encouraged to complete the questionnaire to help improve nursing status and power in the health system.

### 2.3. Data collection

To collect the data, a researcher-made questionnaire was designed and prepared by the researcher based on scientific resources, manuals, and executive guidelines. The questionnaire designed based on of the official duties of professional nurses in Iran. In addition, the research team used theirs many years academic and practical experience in various areas of health care, scientific documents and resources to design the questionnaire. This questionnaire was consisted of two parts: the first part had 10 questions about demographic characteristics and history of attending education courses; the second part included a list of 54 questions that covered the tasks that a nurse could take. Using the Likert scale, there were three options for each question, including: agree, no opinion, and disagree. It should be noted that the main questionnaire was consisted of six sub-sections each indicating a role: care with 16 questions, support with 9 questions, prevention with 12 questions, management with 9 questions, counseling with 4 questions, and education with 4 questions. The study's focus was on nurses roles when to be a member of the physician team. In order to reduce the bias in completing to the questionnaire, the items of the questionnaire were organized and classified into the aforementioned six categories, only after completing the questionnaire by the participants. To prove the face and content validity of the of the questionnaire, it was presented to 10 faculty members who had at least 8 years teaching nursing management and leadership or community health nursing, meanwhile they had above of 5 year experience in health care fields as instructor, manager or professional nurse. Questioner was revised on the basis of theirs comments and suggestions and discriminant validity used for construct validity. Reliability of the tool was evaluated and confirmed by Cronbach's Alpha that totally was 0.93 and for subscales contain of support (0.89), care (0.94), management (0.92), counseling (0.90), prevention (0.91), and education (0.93).

After carrying out the preparatory steps and supplementary studies, a list of the names of the nurses was obtained from the Iranian Nursing Organization and Semnan University of Medical Science. By coordination with hospital and nursing managers in hospital, samples randomly selected based on the list of nurses working in each hospital. Furthermore, researchers visited the nurses at their work place and distributed the questionnaires among them. The nurses could complete the questionnaire in the presence of the researcher or return to a research team member within 24 hours. By coordinating with to collect data from those who were not present at their workplace or were not employed, the researchers made phone calls to set an appointment, and if the contacted person consented to participate in the study, he or she received the questionnaire to complete it. Questionnaire response rate was 75%.

### 2.4. Ethics consideration

All the legal requirements were fulfilled and an ethic approval for the research project was obtained from by the Ethics Committee of Semnan University of Medical Sciences (IR. SEMUMS. REC.1392.606). In addition, the participants were informed about the research goals and received some details about how to complete the questionnaire. They were assured about and the confidentiality of the information and the questionnaire was completed.

### 2.5. Statistics

After collecting the data, they were entered into a computer and analyzed by SPSS 16 software.

## 3. Results

**Figure 1.**
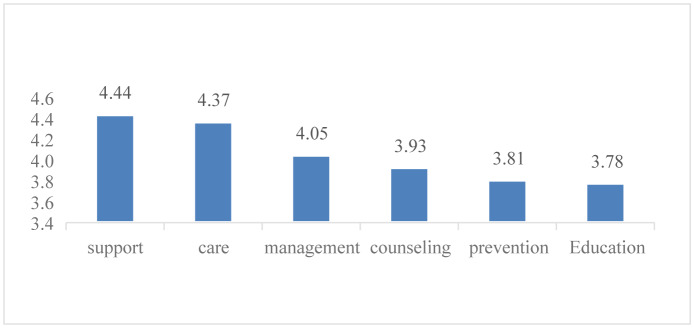
Preferred roles by the nurses in the family physician team.

Of the total number of the desired sample size, 150 nurses participated in this study and completed the questionnaire. Of all, 30 persons (20%) were male and the rest were female (80%). The mean age of the participants was 34.4 years (22 to 55 years). The analysis of collected data using Friedman test showed that the nurses participating in the study were willing to take following roles in the family physician team, arranged from the most favorite to the least favorite: support (4.44), care (4.37), management (4.05), counseling (3.93), prevention (3.81), and education (3.78) (Figure 1).

Spearmen statistical test was used to determine the relationship between occupational experience and the preferred roles. Based on the results, there was no significant relationship between occupational experience and the preferred roles, and even those with more occupational experience had a lower level of tendency in the role of education (Table 1).

**Table 1.** Relationship between job experience and preferred roles of nurses in the family physician team.

Spearman's rho		Prevention	Care	Support	Management	Counseling	Education
Occupational experience	Correlation coefficient	−0.064	0.058	−0.070	0.063	0.092	−0.144
	Sig (2-tailed)	0.444	0.489	0.404	0.456	0.278	0.087
	N	150	150	150	150	150	150

Pearson and Chi square tests were used to determine the relationship between gender and the preferred roles (care, support, prevention, management, counseling, and education). The findings of the tests are presented below (Table 2).

Based on the results of Chi square test, there was a significant relationship between gender and the preferred role of education (p = 0.03). In other words, female nurses have a greater tendency toward undertaking education roles. However, there was no significant difference between the two genders in terms of the other preferred roles (p > 0.05).

Spearman statistical test was used to determine the relationship between age and the preferred roles (care, support, prevention, management, counseling, and education), and the results showed no significant relationship between age and the preferred roles of nurses in the family physician program. Moreover, the roles of prevention and education had a negative relationship with age. In other words, nurses who were older had a lower tendency toward the role of prevention (Table 3).

**Table 2.** Relationship between gender and preferred roles of nurses in family physician team.

		Gender		Total
		Male	Female	
Preferred role of care P = 0.557	Disagree	0	2	2
	No opinion	9	48	57
	Agree	20	71	91
Total		29	120	149
Preferred role of support P = 0.778	Disagree	0	2	2
	No opinion	15	63	78
	Agree	14	55	69
Total		29	120	149
Preferred role of prevention P = 0.773	Disagree	0	2	2
	No opinion	10	42	52
	Agree	19	76	95
Total		29	120	149
Preferred role of management P = 0.657	Disagree	0	2	2
	No opinion	12	56	68
	Agree	17	62	79
Total		29	120	149
Preferred role of counseling P = 0.772	Disagree	0	2	2
	No opinion	15	59	74
	Agree	14	59	73
Total		29	120	149
Preferred role of education P = 0.03	Disagree	1	1	2
	No opinion	21	59	80
	Agree	7	60	67
Total		29	121	150

**Table 3.** Relationship between age and preferred roles of nurses in family physician team.

Spearman test		Preferred roles					
		Prevention	Care	Support	Management	Counseling	Education
Age (year)	Correlation coefficient	−0.092	0.021	−0.12	0.007	0.013	−0.16
	Sig (2-tailed)	0.271	0.798	0.151	0.935	0.88	0.054
Number		150	150	150	150	150	150

## 4. Discussion

Determining job positions and roles precisely is considered as one of the important pillars of human resources organization. 75% of nurses answered to questioner that is acceptable according to standards [25]. One of the important case of refuse complete the questioner by others maybe low familiar and experience with all aspect of theirs professional roles. The findings of the study indicated that the nurses participating in the research tended to be involved in support, care, management, counseling, prevention, and education roles, respectively. In other words, their tendency toward the role of support and care was more than their tendency toward the roles of prevention and education; in addition, they showed a moderate level of tendency toward the roles of management and counseling. A study in Iran showed that nurses' willingness to provide community-based services slightly is in declining due to several reasons such as low support, responsibility for the delivery of out-of-hospital services, and the availability of job vacancies in medical settings [26]. Contrary to the results of the current study, the results of studies in other countries show that nurses, not only play a role in the delivery of health care services, but also play an important role in increasing the knowledge and skills of service recipients [27],[28]. A study by Hopkins and Irvine in the UK (2009) was conducted to explain the nurses' perceptions of the professional roles of specialist nurses in the epilepsy care teams and the methods to face the related challenges. The findings show that nurses are the only professional group that has a comprehensive view toward the fulfillment of patients' needs, but the changes is policies and payment system have somewhat weakened their position. The elimination of nurses form the team, at first, can help to save money, but in a long run, it will lead to inequality in the delivery of services and increase the costs in other sectors. Therefore, the most important solution is to help nurses to prove their role in taking care of these patients [13].

In the present study, the tendency of nurses to play a supportive role is considered as a positive factor and is in line with the results of other studies that highlighted the comprehensiveness of the roles [22] and some studies introduced models for increase effectiveness of advocacy and supportive in health intervention [29]. However, nurses tendency to play the role of care suggests that nurses tend to play the roles in the family physicians team, which they have experienced during many years of activity in hospitals and health care facilities. In line with the results of the present study, the results of a study which investigated and clarified the general roles and capabilities of family nurses (FP-RNs) showed that nurses should distinguish between care services for the family and acute care services provided in the intensive care unit [22].

The relative tendency of nurses participating in the present study toward managerial roles is promising; however, it is necessary to enhance their individual preparedness and legal frameworks as well. It is even believed that nurses can play a role in policymaking [30] and even financial management in different areas of health system [31]. A study by Jane-Badia et al. in Catalonia, which aimed at evaluating the outcomes of the activities performed by the family physician team, show that notice to combination of outcomes helps to assess the performance of family physician team more accurately. The findings of the mentioned study showed that, based on the collected data, the appropriate model had three dimensions: 1) special access to services and facilities and appropriate relationship between staff and patients; 2) coordination between team members; and 3) quality of technical services [32]. Moreover, a study carried out in Central and Eastern Europe (CEE) highlighted key issues such as operationalizing activities, strengthening empowerment plans, and promoting teamwork among the members of the family physician team [23]. Team coordination is an issue which refers to the members within the family physician team and the other groups providing community-based health services, as well. Desmedet and Michel conducted a study aimed at examining the viewpoints of family physicians about how to provide services by a specialist team in charge of the delivery of maintenance care services. The results indicated the appropriateness of medical equipment and facilities, technical competence, and medical support. The lowest level of satisfaction was related to the information received from the team and the identification of the staff as the primary caregivers [33]. The results of other studies on the quality of services provided by the family physician team show that weakness in team coordination is one of the main problems faced by the teams [34],[35]. Therefore, the relative tendency of nurses toward management and teamwork can be considered as a positive factor, which helps improving the team's capabilities and cooperation between nurses in the family physician team.

Since the family physician program is developed with a preventive approach, aimed to promote health and reduce the burden of diseases, the low tendency of nurses toward education and counseling roles is somewhat alarming. It indicates that the nurses are not prepared enough or have an inadequate level of motivation to accept the mentioned roles [36]. On the contrary, the results of other studies indicate the positive effects of the education role of nurses on the status of patients and families [22]. Homaie Rad et al., study in Iran also showed low infrastructure and staffs readiness and proposed to reinforce them [36].

The current study expressed the views of nurses abut theirs role in family physician team. Further studies are recommended to reflect the family physician program directors and other family physician team members such as doctors' viewpoint to better organizing and role distribution.

## 5. Conclusion

The results of the study showed that the nurses were more interested in playing a role in supporting and caring for patients, rather than participating in education and preventive activities. Since family physician teams must mainly provide community-based preventive services, the authorities should try to enhance the interests and capabilities of nurses to empower them to play a role in preventive and education activities.
